# Tyro3 promotes the maturation of glutamatergic synapses

**DOI:** 10.3389/fnins.2024.1327423

**Published:** 2024-02-12

**Authors:** Sheng Miao, Lawrence Fourgeaud, Patrick G. Burrola, Shani Stern, Yuhan Zhang, Kaisa E. Happonen, Sammy Weiser Novak, Fred H. Gage, Greg Lemke

**Affiliations:** ^1^Molecular Neurobiology Laboratory, The Salk Institute for Biological Studies, La Jolla, CA, United States; ^2^Laboratory of Genetics, The Salk Institute for Biological Studies, La Jolla, CA, United States; ^3^Sagol Department of Neurobiology, University of Haifa, Haifa, Israel; ^4^Division of Biological Sciences, University of California, San Diego, La Jolla, CA, United States; ^5^Waitt Advanced Biophotonics Laboratory, The Salk Institute for Biological Studies, La Jolla, CA, United States

**Keywords:** synaptic maturation, receptor tyrosine kinase, phosphatidylserine, GluA2, Hebbian learning

## Abstract

The receptor tyrosine kinase Tyro3 is abundantly expressed in neurons of the neocortex, hippocampus, and striatum, but its role in these cells is unknown. We found that neuronal expression of this receptor was markedly up-regulated in the postnatal mouse neocortex immediately prior to the final development of glutamatergic synapses. In the absence of Tyro3, cortical and hippocampal synapses never completed end-stage differentiation and remained electrophysiologically and ultrastructurally immature. *Tyro3^−/−^* cortical neurons also exhibited diminished plasma membrane expression of the GluA2 subunits of AMPA-type glutamate receptors, which are essential to mature synaptic function. Correspondingly, GluA2 membrane insertion in wild-type neurons was stimulated by Gas6, a Tyro3 ligand widely expressed in the postnatal brain. Behaviorally, *Tyro3^−/−^* mice displayed learning enhancements in spatial recognition and fear-conditioning assays. Together, these results demonstrate that Tyro3 promotes the functional maturation of glutamatergic synapses by driving plasma membrane translocation of GluA2 AMPA receptor subunits.

## Introduction

The three members of the TAM family of receptor tyrosine kinases (RTKs) – Tyro3, Axl, and Mer – were discovered in the early 1990s (Lai and Lemke, 1991; O'Bryan et al., 1991; Graham et al., 1994; Lai et al., 1994). Since that time, a great deal has been learned about the important biological functions of the latter two receptors, which are prominently expressed in macrophages, dendritic cells, and other sentinel cells of the immune system (Lu and Lemke, 2001; Scott et al., 2001; Rothlin et al., 2007; Zagorska et al., 2014; Lemke, 2019). In these cells, Axl and Mer are critical mediators of the phagocytic engulfment and turnover of apoptotic cells (Scott et al., 2001; Zagorska et al., 2014; Lemke, 2019) and concomitantly, of the intrinsic feedback inhibition of Toll-like receptor and cytokine receptor signaling at the end of the innate immune response (Rothlin et al., 2007, 2015). Tyro3 is only modestly expressed by most immune sentinels (Imm Gen, 2016), but is instead prominently expressed by maturing and mature neurons in the central nervous system (CNS) (Lai and Lemke, 1991; Prieto et al., 2007). The roles that Tyro3 might play in these cells, which do not express Axl or Mer, have proven difficult to decipher (Pierce et al., 2008; Zhong et al., 2010; Blades et al., 2020).

As a family, the TAM receptors exhibit unusual signaling properties relative to other RTKs (Lemke, 2013). Their tyrosine kinase enzymatic activities are stimulated by the binding of two closely-related soluble ligands – Gas6 and Protein S (Pros1) (Stitt et al., 1995) – to the receptor ectodomains (Lemke, 2013, 2019). Gas6 activates all three receptors, whereas Pros1 activates Tyro3 and Mer but not Axl (Lew et al., 2014). Pros1 also has important activity as an anticoagulant in the blood coagulation cascade (Burstyn-Cohen et al., 2012), but Gas6 only signals through the three TAM receptors (Lemke, 2013). In addition to ligand binding, full TAM activation also requires the Ca^2+^-dependent binding of phosphatidylserine (PtdSer), a universal membrane phospholipid, to the amino-terminal ‘Gla’ domains of the ligands (Lew et al., 2014). For example, a Gla-less truncation variant of Gas6 binds Axl with the same sub-nanomolar K_D_ as full-length Gas6, but is entirely incapable of activating the Axl tyrosine kinase (Lew et al., 2014). In this respect, TAM receptors and their ligands together act as detectors for PtdSer (Lemke, 2017). This phospholipid is normally sequestered to the cytoplasmic leaflet of plasma membrane bilayers by the action of ‘flippases’ (Segawa et al., 2014, 2016), but is translocated to the extracellular leaflet through the activity of two large families of PtdSer ‘scramblases’ (Suzuki et al., 2010, 2013a; Lemke, 2019). These scramblases are activated by caspase cleavage during apoptosis or alternatively, by calcium binding when cells are depolarized. How these special features of Tyro3 signaling might impact its activities in neurons is also unknown.

We have used cell biological and electrophysiological analyses of cortical and hippocampal neurons from wild-type (WT) versus Tyro3-deficient (*Tyro3^−/−^*) mice (Lu et al., 1999) to investigate Tyro3 activities in these cells. We found that in the absence of Tyro3, glutamatergic synapses were both physiologically and ultrastructurally immature. Correspondingly, we observed that Gas6 activation of post-synaptic Tyro3 in WT neurons stimulated the essential plasma membrane insertion of GluA2 AMPA receptor subunits at synapses. We further found that this stimulation was dependent on plasma membrane externalization of PtdSer, which is induced by pre-synaptic depolarization, as Gla-less Gas6 was without effect. Finally, *Tyro3^−/−^* mice exhibited behavioral and learning enhancements that are consistent with unusual synaptic plasticity. Together, these observations identify Tyro3 as a mediator of post-synaptic maturation events that are triggered by the pre-synaptic display of phosphatidylserine.

## Materials and methods

### Mice

C57BL/6 wild-type mice were obtained from The Jackson laboratory. The *Tyro3*^−/−^ strain has been described previously (Lu et al., 1999). All animal procedures were conducted in compliance with the ethical guidelines and according to protocols approved by the Salk Institute Animal Care and Use Committee (IACUC) (Protocol Nos. 15-0001, 15-0079, and 17-0046).

### Hippocampal and cortical neuron culture

Hippocampal and cortical neurons were prepared according to previously described methods, with several modifications (Hilgenberg and Smith, 2007; Beaudoin et al., 2012). Briefly, hippocampi or cortices from embryonic day 18 (E18) fetuses from timed-pregnant C57BL/6 mice were micro-dissected and chopped into small pieces in Hank’s Balanced Salt Solution (HBSS) without Ca^2+^/Mg^2+^, with 10 mM HEPES, and then treated with a digestion solution containing HBSS with Ca^2+^/Mg^2+^, 10 mM HEPES, 20 units/ml papain and 0.005% DNase for 30 min at 37°C. After digestion, tissues were mechanically dissociated using a pasteur pipette (with tip fire-polished) in HBSS with Ca^2+^/Mg^2+^, 10 mM HEPES and trypsin inhibitor. Cell suspensions were plated at 15,000 cells/well on poly-L-lysine precoated glass coverslips (Neuvitro) in 24-well plates for immunocytochemistry, at 150,000/well on poly-L-lysine precoated glass coverslips (Neuvitro) in 24-well plates for electrophysiological recording, and at 10^6^/dish in PLL-coated 35 mm dishes for biochemistry. The cells were maintained in plating media containing Neurobasal™ Plus Medium (Thermo Fisher Scientific), 2% B-27™ plus supplement (Thermo Fisher Scientific), 1% GlutaMAX™ supplement (Thermo Fisher Scientific) and 5% heat inactivated FBS for 3 h. After attachment of the cells, the media was switched to culturing media, comprised of Neurobasal™ Plus Medium, 2% B-27™ plus supplement and 1% GlutaMAX™ supplement. From 4 days-*in-vitro* (DIV) onwards, half medium changes were performed with Culturing medium. To inhibit proliferation of glial cells, 0.5 μM cytosine arabinoside (AraC, Sigma-Aldrich) was added to the medium at 4 DIV. Cultures were maintained in a humidified incubator at 37°C and 10% CO_2_.

### Hippocampal slice electrophysiology

Acute coronal vibratome slices (350 μm) of postnatal day (P)30-P50 (for field recordings) and P14-16 (for whole cell recordings) C57Bl/6 J mouse hippocampi were prepared and analyzed using standard protocols (Lein et al., 2011), as described previously (Fourgeaud et al., 2010). Briefly, mice were anesthetized with isoflurane inhalation and decapitated, and slices were cut into ice-cold cutting solution (in mM: 85 NaCl, 75 sucrose, 2.5 KCl, 0.5 CaCl_2_, 4 MgCl_2_, 1.25 NaH_2_PO_4_, 25 NaHCO_3_, 25 glucose, and 2.25 ascorbate) equilibrated with 95% O_2_/5% CO_2_. Slices were incubated 30 min at 30°C in oxygenated artificial cerebrospinal fluid (ACSF) (in mM: for whole-cell recording experiments, 126 NaCl, 2.5 KCl, 1.25 NaH_2_PO_4_, 2 MgCl_2_, 2 CaCl_2_, 26 NaHCO_3_, and 18 glucose; for field recording experiments, 126 NaCl, 2.5 KCl, 1.25 NaH_2_PO_4_, 1.3MgSO_4_, 2.5CaCl_2_, 26 NaHCO_3_, and 10 glucose) and maintained at room temperature at least 1 h before being transferred to a submerged recording chamber.

Whole-cell patch-clamp recordings of excitatory postsynaptic currents (EPSCs) from CA1 pyramidal neurons were conducted at room temperature. Picrotoxin (50 μM) was added to the ACSF to block GABA_A_ receptor-mediated inhibition, and connections between CA3 and CA1 were cut to reduce epileptiform activity. Patch electrodes (3–5 MΩ) were filled with intrapipette solution [in mM: 108 cesium gluconate, 20 Hepes, 0.4 EGTA, 2.8 NaCl, 5 TEACl, 4 MgATP, 0.3 NaGTP, 10 phosphocreatine, adjusted to pH 7.2 with CsOH (290 mosM)]. CA1 pyramidal cells were voltage-clamped at −70 mV, and spontaneous EPSCs (sEPSCs) were recorded. To record mini EPSCs (mEPSCs), 0.5 μM TTX was added to the ACSF.

Field recordings from populations of CA1 neurons were conducted at room temperature using standard methods. Picrotoxin (100 μM) was added to the ACSF to block GABA_A_ receptor-mediated inhibition and connections between CA3 and CA1 were cut to reduce epileptiform activity. Stainless-steel bipolar electrodes were used to stimulate Schaffer collateral fibers (0.05 Hz), and field excitatory postsynaptic potentials (fEPSPs) were recorded from stratum radiatum using extracellular glass microelectrodes (3–5 MΩ) filled with ACSF. Input–output (I/O) relationships were determined by measuring fEPSP (output) amplitudes in relation to stimulation intensity (input). After establishing a stable 20 min baseline, LTP was induced using a high frequency stimulation (HFS) protocol (100 Hz, 1 s duration at test strength). Data are represented normalized to the average fEPSP amplitude 10 min before HFS.

### Whole-cell patch-clamp recordings from hippocampal neuronal cultures

Cultured hippocampal neurons (13–14 DIV) were prepared as described above. Neurons on glass coverslips were transferred to a recording chamber in standard artificial cerebrospinal fluid (ACSF) containing 10 mM HEPES, 4 mM KCl, 2 mM CaCl_2_, 1 mM MgCl_2_, 139 mM NaCl, 10 mM d-glucose (osmolarity adjusted with sucrose to 320 mOsm, and pH adjusted to 7.4). Cells were patch-clamped with pipettes containing 130 mM K-gluconate, 6 mM KCl, 4 mM NaCl, 10 mM Na-HEPES, 0.2 mM K-EGTA, 0.3 mM GTP, 2 mM Mg-ATP, 0.2 mM cAMP, 10 mM D-glucose, 0.15% biocytin and 0.06% rhodamine, pH 7.2 (pipette tip resistance was 5–8 MΩ). Action potentials were evoked by injecting depolarizing current pulses in current-clamp mode. Spontaneous EPSCs were recorded in voltage-clamp at −60 mV with a 20 kHz sampling rate with inhibitory activity blocked by 40 μM bicuculline. Signals were amplified with a Multiclamp700B amplifier and recorded with Clampex 10.4 software (Axon Instruments). Miniature EPSCs were recorded also in voltage-clamp mode but with the addition of 0.5 μM TTX. Data were subjected to a 3,000 Hz low-pass filter and analyzed using Clampfit-10 and the software package Matlab (2014A, The MathWorks Inc., Natick, MA, 2000). All measurements were conducted at room temperature.

### Cortical neuron treatments

Primary cortical neurons were cultured for 11 DIV before starting treatment with either 20 nM Gas6 or 20 nM Gla-less Gas6. For 7 day long-term treatment, cortical neurons were treated starting at 11 DIV with supplement every other day when medium was half changed. For 1 day-short term treatment, 20 nM Gas6 were added only once to cultures at 16 DIV with or without pretreatment using 100 nM Purified Recombinant Annexin V (BD Pharmingen, #556416). Treatment with TBS buffer, the solvent for Gas6, were used as negative control. To visualize exposed phosphatidylserine on plasma membrane during neuron development *in vitro*, cortical neurons at 2, 5, 8, 12, 16, or 20 days *in vitro* (DIV) were incubated with pSIVA-IANBD (Novus Biologicals, NBP2-29382) diluted to 20 μl/ml and CellTracker Red CMTPX dye (Invitrogen C34552) at 0.5 μM in culture medium for 60 min at 37°C, 10% CO_2_ before fixing with 4%PFA. To stain exposed phosphatidylserine after calcium ionophore A23187 treatment, cortical neurons at 18 DIV were treated with A23187 at concentration of 5 μM for 20 min in medium also containing pSIVA-IANBD diluted to 20 μl/ml and 0.5 mM CellTracker Red CMTPX dye before fixing with 4% PFA.

### GluA2 live staining in mouse cortical neurons

Mouse cortical neurons were live stained for GluA2 according to previously described methods (Blanco-Suarez et al., 2018), with several modifications. Briefly, cortical neurons on coverslips were incubated with mouse anti-GluA2 (Millipore MAB397) at a 1:100 dilution and CellTracker Red CMTPX dye (Invitrogen C34552) at a 0.5 mM concentration in culture medium for 30 min at 37°C, 10% CO_2_. After washing with DPBS and fixing with pre-warmed (34°C) 4% PFA for 5 min, the coverslips were washed with PBS and then blocked (without permeabilizing) with PBS containing 2% BSA and 5% normal goat serum for 1 h at room temperature in a humidified chamber. After blocking, coverslips were incubated with goat anti-mouse Alexa 488 at 1:500 dilution in PBS containing 2% BSA and 5% normal goat serum for 3 h at room temperature. Coverslips were then washed with PBS and mounted in Prolong Diamond antifade mounting medium with DAPI (Molecular Probe REF P36962). Neurons were imaged on Zeiss LSM700 using the 63x oil-immersion objective. At least 45 cells per condition were imaged across three coverslips in each independent experiment. A minimum of 3 independent experiments were run. Cells were located using CellTracker Red CMTPX in the red channel. Exposure acquisition was set according to neurons with Gas6 treatment within each independent experiment, capturing 16 bit images at a resolution of 1024×1024 pixels. GluA2 puncta on the soma and dendrites of cortical neurons were analyzed using Imaris software and normalized to cell volume that was calculated based on CellTracker Red signals.

### Perfusion and tissue processing for electron microscopy

Mice were anesthetized with 2.5% Avertin/Saline IP, and then dissected open from the abdomen up into the chest cavity. The right atrium was nicked and perfusion was performed through the left ventricle with the following solutions: 1 × 1′ Ringers at 37°C, 1 × 9′ 2.5% glut/2.0% Para/1 mM CaCl_2_/ 0.15 M Caco. pH7.3 at 37°C. The brain was cut with a razor blade in a coronal plain using the “brain jig.” Cuts were made at 4 mm anterior and 5 mm posterior in the brain jig. First fixing was performed for 2 h at 4°C in 2.5% glut/2.0% Para/1 mM CaCl_2_/0.15 M Caco. pH7.3. The brain was embedded in 2.5% agar/0.15 M Caco then mounted with super glue on a stub for cutting. Coronal sections (300 μm) were cut through the brain in pairs. Sections were placed in a 12 well plate with 0.15 M caco buffer pH 7.3. Sections were washed overnight in 0.15 M caco buffer pH 7.3 at 4°C, and slices were trimmed down to the specific hippocampus area of interest in the CA1 region just above dentate gyrus. In a second fixing, the trimmed sections were incubated in 2% Osmium Tetroxide/0.15 M caco. pH7.3 for 3 h on ice in a fume hood, followed by 5 times washing in milli-Q water on ice. A third fixation was performed in 2% Uranyl Acetate for 45 min on ice. Dehydration was performed in 30, 50, 70, 95% ETOH (in sequence) for 8 min each and in 100% ETOH twice for 8 min each, and then in acetonitrile. Infiltration was performed in 66/33 ratio of resin/acetonitrile for 30 min, in Epon/Araldite for 2 h at room temperature (RT), and then in Epon/Araldite for 3 h at RT. Embedding was performed in fresh resin using flat embedding capsules at 65°C overnight.

### Electron microscopy image analyses

**Figure 1:**
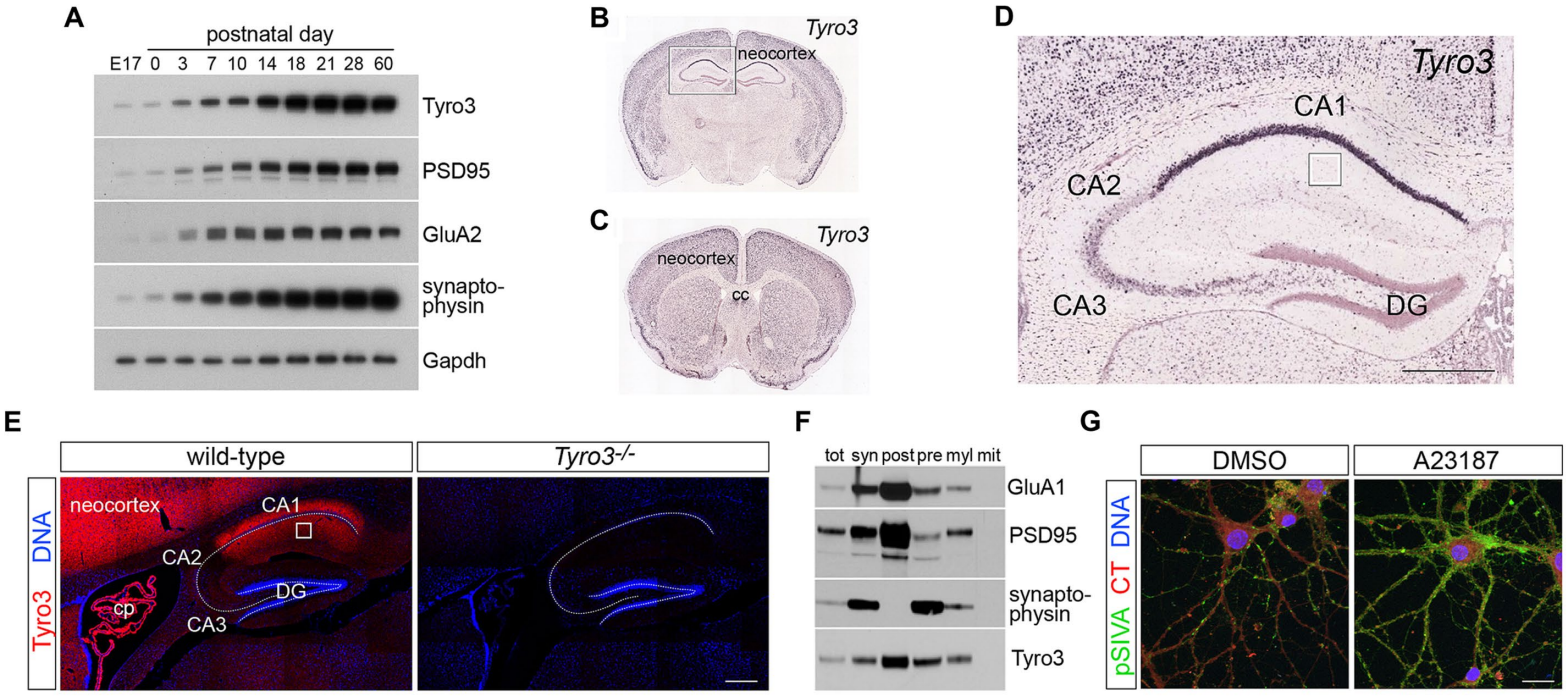
Expression of Tyro3 system components in the CNS. **(A)** Representative western blot analyses of the expression of the indicated proteins in the mouse neocortex at the indicated developmental times. Gapdh is a loading control. **(B–D)** Images from the Allen Brain Atlas (http://mouse.brain-map.org/gene/show/21931) of expression of *Tyro3* mRNA in the postnatal day (P) 56 mouse brain, as monitored by *in situ* hybridization of digoxigenin riboprobes to coronal sections (Jones et al., 2009). Boxed region in **(B)** is enlarged in **(D)**. Boxed region in **(D)** is the stratum radiatum of the hippocampus, the area analyzed by electron microscopy in Figure 3. **(E)** Tyro3 protein expression (red), detected with a Tyro3 antibody (Prieto et al., 2007), in adult WT versus *Tyro3^−/−^* (antibody control) hippocampus. DAPI (blue, DNA) labels nuclei. **(F)** Density gradient membrane fractionation of P30 mouse hippocampus, analyzed by western blot for the indicated proteins. **(G)** Representative images of cultured cortical neurons (see Methods) at 18 DIV treated for 20 min with di-methyl sulfoxide (DMSO, vehicle control, left) or the calcium ionophore A23187 (5 μm, right) and simultaneously incubated with pSIVA (green) to visualize externalized phosphatidylserine (PtdSer) on the plasma membrane surface and CellTracker Red to delimit cells. DAPI (blue, DNA) labels nuclei. E17, embryonic day 17; cc, corpus callosum; DG, dentate gyrus; cp, choroid plexus; tot, total cortical protein; syn, synaptosomes; post, post-synaptic membrane; pre, pre-synaptic membrane; myl, myelin; mit, mitochondria; CT, CellTracker dye. Scale bars: **(D,E)** 100 μm; **(G)** 20 μm.

Synapses were defined by the presence of a stained postsynaptic density facing (apposed to) a presynaptic process containing at least three synaptic vesicles. Perforated synapses were defined by the presence of a discontinuity in the postsynaptic density (Geinisman et al., 1987), and multiple synapse boutons (MSBs) were defined by the presence of at least 2 independent dendritic spines contacting the same axon terminal (Sorra and Harris, 1993). Three sections were analyzed per block, and 2 blocks per animal were used to collect micrographs. Transmission electron microscopy (TEM) micrographs were taken on a Zeiss Libra 120 microscope operated at 80 kV, using a Gatan Ultrascan 4,000 camera at 4 K resolution at an initial magnification of 4,000×, and enlarged photographically to a final magnification of 21,700×. Synapses were randomly photographed in an area corresponding to the stratum radiatum (boxed in Figure 1D), the suprapyramidal region of the hippocampus that contains CA3-to-CA1 Schaffer collaterals. The density of perforated synapses and MSBs were estimated by two independent observers who were blind to genotype.

Volumetric data from serial sections in the scanning electron microscope (SEM; S3EM) were also collected. Briefly, resin embedded samples from control and *Tyro3^−/−^* conditions were selected, the stratum radiatum was identified from semi-thin sections, and a frustum of 150 × 400 × 60 μm was trimmed using a Leica UC7 ultramicrotome. A silicon chip (35 × 7 mm, University Wafer, Boston, MA) was glow discharged in a plasma cleaner (Harrick, Ithaca), rinsed in deionized water, and partially immersed in a Diatome Histo knife. An ionizing gun (Leica EM Crion) was activated and oriented toward the cutting edge of the knife mounted on the ultramicrotome. Ribbons of approximately 100 serial sections of 50 nm thickness were cut, the water level was slowly lowered, and sections were allowed to dry on the silicon. Samples were mounted using carbon sticky tabs and loaded into a Zeiss Sigma VP SEM, and serial sections were imaged using the array tomography software module of Atlas5 (FIBICS, Ottawa). A region of interest unobstructed by artifact throughout the series was identified and 2 nm/px images were collected on consecutive sections. Images were aligned using rigid and affine alignment functions available in TrakEM2 in Fiji. Evaluation of datasets and segmentation of dendritic spines was conducted in Reconstruct. The serial 2D segments were exported as VRML mesh files and imported into Blender software^1^ for further visualization.

### Subcellular fractionation

Subcellular fractions of P30 hippocampus were obtained using established protocols (Blackstone et al., 1992), as described previously (Fourgeaud et al., 2010). Briefly, animals were anesthetized with isoflurane inhalation and decapitated, brains were quickly removed, and hippocampi were dissected out and homogenized in 10 vol of Hepes sucrose buffer (0.32 M sucrose and 4 mM Hepes, pH 7.4) supplemented with protease inhibitor mixture (Complete, Roche) using a glass- Teflon homogenizer at 900 rpm (12 strokes). The homogenate was centrifuged for 10 min at 1,000 × *g*, and the pellet (P1) containing the nuclei was discarded. The supernatant (S1) was centrifuged for 20 min at 10,000 × *g*, resulting in a crude synaptosomal pellet (P2). P2 was resuspended in 10 vol of Hepes sucrose buffer and centrifuged for 10 min at 20,000 × *g*, resulting in a washed crude synaptosomal pellet (P2′). P2′ was lysed by hypo-osmotic shock in water and adjusted to 4 mM Hepes. After 30 min of gentle rotation at 4°C, P2′ was centrifuged for 20 min at 25,000 × g, resulting in a crude synaptosomal membrane fraction (P3) and a crude synaptic vesicle fraction (S3). Aliquots were collected at multiple steps of the fractionation and solubilized in Triton X-100 (1%) for Western blot analysis. PSD-enriched fractions were prepared as described previously (Zhou et al., 2007).

### SDS-PAGE and western blot

Protein samples were prepared with reducing Laemmli buffer and boiled for 10 min at 95°C, and then separated on NuPAGE 4–12% Bis-Tris Mini protein gel (Thermo Fisher Scientific NP0323BOX) for 3 h at constant voltage of 90 V. After gel electrophoresis, proteins were transferred onto Immobilon-P PVDF membranes (Millipore IPVH00010) for 90 min at constant current of 0.4A. Membranes were blocked with 1x Tris Buffered Saline (TBS) with 1% Casein (BioRad, #1610782) for 1 h at room temperature in gentle shaking, and then incubated at 4°C overnight with the primary antibody in blocking buffer, rabbit anti-Tyro3 (Cell signaling 5585S) at a dilution of 1:2000, mouse anti-PSD95 (Thermo Fisher Scientific MA1-046) at a dilution of 1:2000, mouse anti-GluA2 (Millipore MAB397) at a dilution of 1:5000, rabbit anti-synaptophysin (Abcam ab16659), or mouse anti-GAPDH (Santa Cruz SC-32233) at a dilution of 1:10,000. After washing with TBS buffer containing 0.1% Tween-20, membranes were incubated with secondary antibody for 3 h at room temperature (Peroxidase AffiniPure Goat Anti-Rabbit IgG, Jackson Immunoresearch 111–036-047 or 115–006-072). After washing with TBS buffer containing 0.1% Tween-20, membranes were incubated in chemiluminescent substrate (SuperSignal, ThermoFisher 34,580) and the chemiluminescent signal was detected and recorded by exposure of the membrane to X-ray film.

### Open field tests

Mice (male, age 3 months) were placed in a novel open field [16″ (W) x 16″ (D) x 15″ (H)] and allowed to explore it for a period of 20 min. Horizontal and vertical movement were automatically tracked utilizing 2 parallel infrared beam arrays surrounding a Plexiglas arena. The total distance the mice traveled, and percentage time they spent in peripheral and center regions of the open filed were analyzed.

### Morris water maze

In the hidden version of the Morris water maze test (Morris et al., 1982), mice (male, age 3 months) were trained with two blocks per day over 10 days, with each block consisting of two trials with 30 s interval between the trials. The platform was hidden 1 cm below the surface of water made opaque with white nontoxic paint. Starting points were changed every trial and every day. In each trial, mice were given 60 s to find the platform. If mice found the platform earlier than 60 s, the trial ended then. If mice failed to find the platform, the trial terminated at 60s and they were guided to reach the platform. After mice had reached platform, they were allowed to rest on it for 10 s. On the 11th day, the platform was removed for a 60 s probe trial. Swim path length and speed were recorded (Ethovision; Noldus Information Technology, Wageningen, The Netherlands).

### Contextual fear conditioning

Prior to behavioral procedures, mice aged at 3 months were acclimatized to the testing room and handled by the experimenter for 1 week. On the eighth day (experimental day 1), mice were allowed to explore the context A for 10 min. On day 2, the mice were placed into context A for fear conditioning for a total time of 42 s. The mice were firstly permitted 10s to explore the context, and then presented with 2 s 0.7 mA electric shocks to the foot followed by 30s rest before return to home cage. On day 3, freezing behaviors were monitored first in conditioned context A for 5 min, and 90 min later in a novel context B for another 5 min.

### Quantification and statistical analysis

Results are expressed as mean ± s.e.m. Statistical analysis was carried out in Prism 9.1.0 (Graph-Pad, La Jolla, CA, USA). A two-tailed unpaired Student’s *t*-test was used for statistical comparisons between two groups. For analysis of Morris water maze and contextual fear conditioning tests, a two-way repeated measures ANOVA was used. Bonferroni’s multiple comparisons test was used for *post hoc* analyses. Sample sizes were chosen based on previous studies. No statistical methods were used to pre-determine sample sizes.

## Results

### Neuronal Tyro3 expression in the postnatal brain

We first confirmed several unusual features of Tyro3 expression that have been reported previously (Lai and Lemke, 1991; Lai et al., 1994; Ohashi et al., 1994; Prieto et al., 2000; Funakoshi et al., 2002; Prieto et al., 2007). As for the other members of the TAM family, multiple studies have documented only limited expression of Tyro3 in developing mouse and rat embryos. Instead, a substantial up-regulation in the expression of this RTK, which is largely restricted to neurons in the neocortex, hippocampus, and other higher centers of the brain, was detected by western blot in the mouse cortex during the first 2 weeks of postnatal life (Figure 1A; Lai et al., 1994). High Tyro3 expression was maintained in the adult brain (Figure 1A; Lai and Lemke, 1991; Lai et al., 1994). With respect to the results presented below, it is important to note that this time course of cortical up-regulation immediately precedes the time course of the maturation of glutamatergic synapses, as monitored by expression of the post-synaptic scaffolding protein PSD-95, the synaptic vesicle protein synaptophysin, and the GluA2 subunit of AMPA-type glutamate receptors (Figure 1A). The maturation of glutamatergic synapses has previously been shown to be dependent upon the *de novo* plasma membrane insertion of these calcium-impermeable GluA2 subunits, and in the mouse, this occurs between postnatal day (P)7 and P16 (Kumar et al., 2002; Brill and Huguenard, 2008). Although *Tyro3* mRNA has been detected in select immune (Chan et al., 2016; Imm Gen, 2016) and tumor cells (Graham et al., 2014), and in newborn oligodendrocytes (Akkermann et al., 2017), it is overwhelmingly the product of adult neurons in the brain (Prieto et al., 2007; Zhong et al., 2010; Figures 1B,C). While *Tyro3* mRNA is abundant in neurons across all layers of the adult neocortex (Figures 1B,C), and in striatum (Figure 1C) and hippocampus (Figures 1B,D; Lai and Lemke, 1991; Prieto et al., 2000), it is not significantly expressed in the thalamus, hypothalamus, midbrain, or spinal cord.

Neuronal expression of Tyro3 has previously been shown to exhibit a marked disparity across the fields of the adult hippocampus, with very high expression in CA1, very low expression in CA3, and no expression in CA2 (Figure 1D; Lai and Lemke, 1991). [Tyro3 was among the first identified molecular markers that distinguish CA1 neurons in the hippocampus (Lai and Lemke, 1991)]. Staining of adult mouse hippocampal sections with a Tyro3 antibody (Prieto et al., 2000) revealed that Tyro3 protein was abundant in both the apical and basal processes of CA1 neurons (Figure 1E), as has previously been reported for cortical neurons (Prieto et al., 2007). When we performed densitometric biochemical fractionation of membranes from P30 mouse hippocampus (see Methods), we observed that Tyro3 largely, although not exclusively, co-fractionated with post-synaptic rather than pre-synaptic markers (Figure 1F), consistent with its previously observed localization to neuronal dendrites in the neocortex (Prieto et al., 2007). This localization is also consistent with the Tyro3 activation of post-synaptic protein kinase C and other kinases that has been observed in a mouse model of frontotemporal lobar degeneration (Fujita et al., 2018).

As noted above, the Tyro3 tyrosine kinase is activated by two closely-related, secreted (soluble) extracellular ligands – Gas6 and Protein S (Pros1) – both of whose mRNAs are very widely expressed across many different cell types in the CNS (Prieto et al., 1999, 2000). In addition to their expression in neurons, the *Gas6* and *Pros1* mRNAs are abundant in all microglia (Imm Gen, 2016), where they regulate multiple microglial properties (Fourgeaud et al., 2016; Huang et al., 2021), and in brain endothelial cells (Happonen et al., 2023)^2^.

As also noted above, the final component that is essential for TAM receptor activation and signaling is the membrane glycerophospholipid phosphatidylserine (PtdSer) (Lemke, 2013, 2019; Lew et al., 2014). The N-terminal ‘Gla’ domains of Gas6 and Pros1 bind to PtdSer, whereas the C-terminal ‘SHBG’ domains of these ligands bind to the Ig domains of the TAM receptor ectodomains (Lew et al., 2014). In plasma membranes, a set of flippase enzymes (Segawa et al., 2014; Andersen et al., 2016; Segawa et al., 2016) normally confines PtdSer to the inner leaflet of the membrane bilayer (Leventis and Grinstein, 2010), but in neurons and all other cells, PtdSer is translocated to the outer leaflet by the action of both Ca^2+^- and caspase-activated scramblases (Suzuki et al., 2010, 2016; Whitlock and Hartzell, 2017; Lemke, 2019). Effective TAM signaling cannot occur in the absence of PtdSer externalization, as it is the only way in which this phospholipid can access the Gla domains of Gas6 and Pros1 (Lew et al., 2014; Dransfield et al., 2015; Lemke, 2017).

We visualized PtdSer externalization in cultured cortical neurons using pSIVA, a ‘polarity-sensitive indicator for viability and apoptosis’, derived from Annexin B12, which fluoresces strongly only when bound to PtdSer (Kim et al., 2010; Huang et al., 2021). Neurons were isolated from embryonic day (E)18 cortices, and cultured *in vitro* using established protocols (Hilgenberg and Smith, 2007; Beaudoin et al., 2012). As has been described previously (Lesuisse and Martin, 2002; Beaudoin et al., 2012), we found that these cells underwent maturation over the first 14 days *in vitro* (DIV), marked by the up-regulation of Tyro3, GluA2, and PSD-95 by 11 DIV (Supplementary Figure S1A). This *in vitro* time course paralleled the expression of these same markers *in vivo* (Figure 1A). Incubation of differentiating cortical neurons with pSIVA revealed that prominent PtdSer externalization occurred between 16 and 20 DIV (Supplementary Figures S1B,C) – when synaptogenesis and spontaneous electrical activity are robust (Lesuisse and Martin, 2002; Priller et al., 2007; Beaudoin et al., 2012). This externalized PtdSer was frequently seen to be localized on neuronal membrane overlying puncta of presynaptic synaptophysin (Supplementary Figure S1D). Spontaneous electrical activity allows for calcium influx and the activation of Ca^2+^-dependent scramblases (Suzuki et al., 2010; Whitlock and Hartzell, 2017), which are the enzymes that translocate PtdSer to the membrane surface. These scramblases can also be artificially activated by calcium ionophores (Suzuki et al., 2010, 2013b). When we strongly activated Ca^2+^ influx in 18 DIV cortical neurons using the calcium ionophore A23187 (Mattson, 1990) (5 μM for 20 min), we triggered massive PtdSer externalization onto the surface of these cells without inducing cell death (Figure 1G).

Together, the above results demonstrate that all of the essential components of TAM signaling – Tyro3, the ligands Gas6 and Pros1, and PtdSer – are mobilized in and around neurons of the maturing postnatal brain. It is important to note that in most settings the tripartite complex of PtdSer-TAM ligand-TAM receptor has been found to link the membranes of two apposed cells (Lemke, 2013, 2017, 2019), with PtdSer on one cell, a TAM receptor on the apposed cell, and a TAM ligand bridged between (Lemke, 2017). As discussed below, it is likely that such a tripartite PtdSer-Gas6-Tyro3 bridging complex links the apposed pre- and post-synaptic membranes of active synapses.

### Immature physiology of *Tyro3*^−/−^ synapses

**Figure 2:**
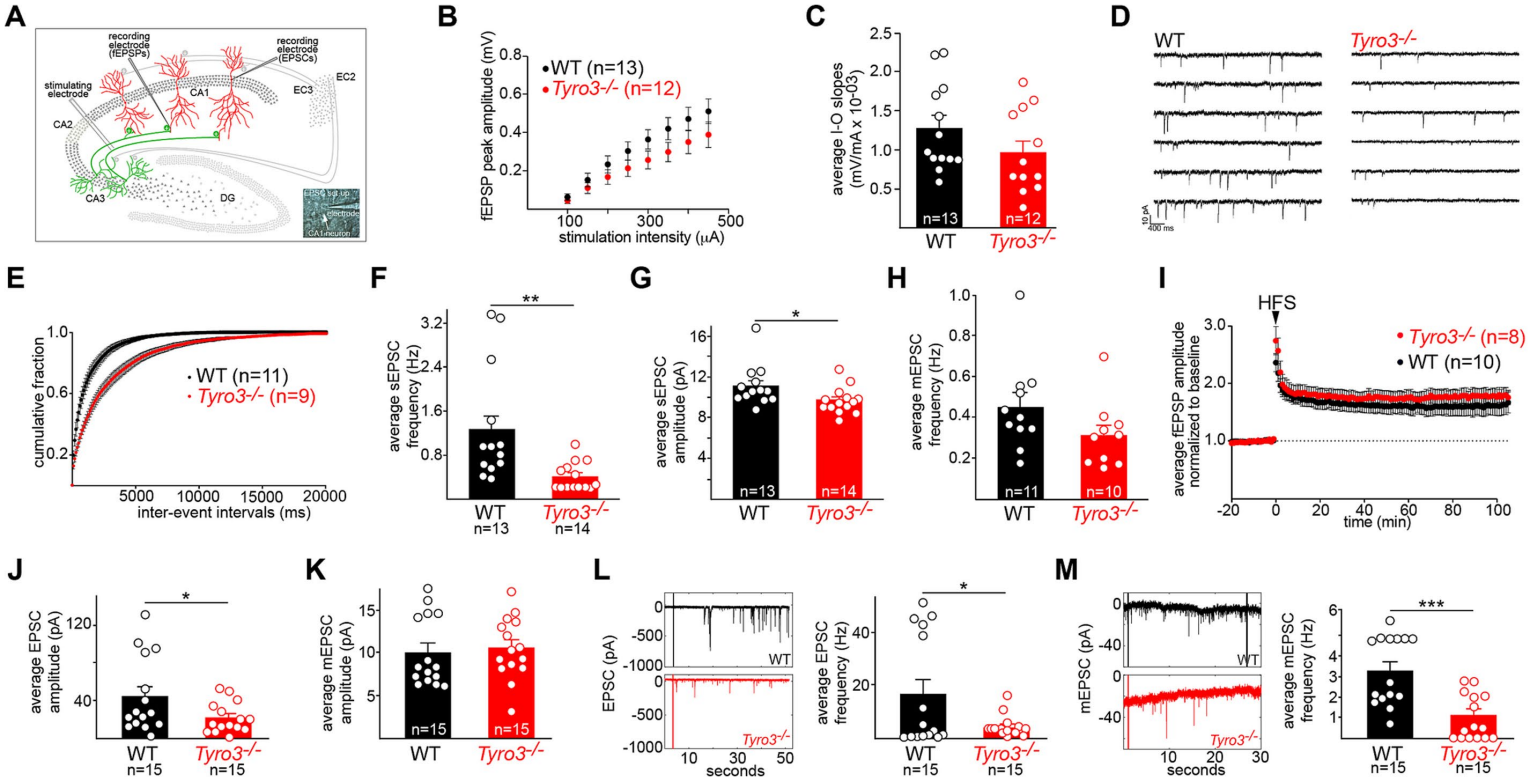
Immature synaptic physiology of *Tyro3^−/−^* neurons. **(A)** Standard *in vitro* recording set-up for mouse hippocampal slices, illustrating the positions of stimulating and recording electrodes within the slice (see Methods). EC, entorhinal cortex. Inset: electrode in a CA1 neuronal cell body. **(B)** Quantification of averaged field excitatory post-synaptic potential (fEPSP) amplitudes (in millivolts, mV) versus stimulus intensity (microamperes, μA) in the indicated number of WT versus *Tyro3^−/−^* hippocampal slices (one slice per mouse). **(C)** Mean of input–output (I–O) slopes for WT versus *Tyro3^−/−^* slices. *n* is number of slices for each genotype; one slice per mouse. **(D)** Representative traces of spontaneous EPS currents (sEPSCs) in WT and *Tyro3^−/−^* slice. **(E)** Cumulative distribution of mean sEPSC inter-event intervals in WT (11 mice) versus *Tyro3^−/−^* (9 mice). **(F)** Quantification of the average frequency of sEPSCs (Hertz, Hz) in the indicated number of WT versus *Tyro3^−/−^* cells. **(G)** Quantification of the average amplitude of sEPSCs (pA) in the indicated number of WT versus *Tyro3^−/−^* cells. For panels **(F,G)**, *p*-values are * <0.05, ** < 0.01, Mann–Whitney test. **(H)** Quantification of the average frequency of mini EPSCs (mEPSCs), recorded in the presence of TTX, in the indicated number of WT versus *Tyro3^−/−^* cells. *n* is number of cells recorded from 7 WT and 5 *Tyro3^−/−^* mice. **(I)** Induction of hippocampal LTP by high frequency stimulation (HFS; see Methods) in WT versus *Tyro3^−/−^* hippocampal slices. *n* is number of slices for each genotype; one slice per mouse. Average fEPSPs are normalized to 10 min baseline. **(J)** Quantification of average EPSC amplitude in picoamperes (pA) in cultured hippocampal neurons prepared from WT versus *Tyro3^−/−^* mice. **(K)** Quantification of average mEPSC amplitude (pA) in cultured hippocampal neurons prepared from WT versus *Tyro3^−/−^* mice. **(L)** (Left) representative traces of EPSCs recorded over 50 s in cultured hippocampal neurons prepared from WT versus *Tyro3^−/−^* mice. (Right) Average EPSC frequency (Hz) in cultured hippocampal neurons prepared from WT and *Tyro3^−/−^* mice. **(M)** (Left) representative traces of mEPSCs recorded over 30 s in cultured hippocampal neurons prepared from WT versus *Tyro3^−/−^* mice. (Right) Average mEPSC frequency in cultured hippocampal neurons prepared from WT versus *Tyro3^−/−^* mice. For panels **(J–M)**, *n* is number of cultured WT and *Tyro3^−/−^* neurons analyzed. *p*-values: * <0.05; ** <0.01, *** <0.001, Mann–Whitney test.

We have previously described the generation and analysis of full germline mouse loss-of-function mutants for *Tyro3* (Lu et al., 1999). *Tyro3^−/−^* mice are born in Mendelian ratios, have normal life spans, are fertile as males and females, and display superficially normal histology in the brain and other tissues (Lu et al., 1999; but see Akkermann et al., 2017). They are full-length protein nulls (Figure 1E; Lu et al., 1999; Lu and Lemke, 2001; Lew et al., 2014; Zagorska et al., 2014). Given the localized expression of Tyro3 in neurons of the hippocampus (Figures 1B,D), we first examined the physiology of the synaptic connection between pre-synaptic CA3 neurons and post-synaptic CA1 neurons – the ‘Schaffer collaterals’ (Szirmai et al., 2012) – in standard *ex vivo* hippocampal slice preparations (Fourgeaud et al., 2010) prepared from male WT versus *Tyro3^−/−^* brains (Figure 2A; see Methods). At this synapse, Tyro3 presumably acts post-synaptically, since it is abundantly expressed on the pyramidal dendrites of CA1 neurons, but not by CA3 neurons (Figures 1B,D,E). We stimulated the Schaffer collaterals and recorded field excitatory postsynaptic potentials (fEPSPs), spontaneous excitatory postsynaptic currents (sEPSCs), and miniature excitatory postsynaptic currents [mEPSCs, recorded in the presence of tetrodotoxin (TTX)] in CA1 (Figure 2A) (see Methods).

When we measured the input–output (I-O) relationship of the fEPSP in WT versus *Tyro3^−/−^* hippocampal slices, we found that both fEPSP peak amplitudes (Figure 2B) and average input–output slopes (Figure 2C) for CA3 to CA1 synapses were smaller in *Tyro3^−/−^* mice, but this difference was not statistically significant. However, when we monitored sEPSCs, which reflect both action potential-dependent and spontaneous neurotransmitter activity at synapses (recordings performed in the presence of 0.5 μM picrotoxin to block GABA_A_ transmission), we measured much more substantial physiological deficits (Figure 2D). The mean inter-event intervals between sEPSCs (Figure 2E) were longer, and the average frequency of sEPSCs (Figure 2F) was markedly reduced in *Tyro3^−/−^* recordings. The average amplitude of sEPSCs was also significantly reduced in the mutants, albeit less so (Figure 2G). When we added TTX to inhibit action potentials and monitor mEPSCs, which report only action potential-independent spontaneous vesicle release, we observed a statistically insignificant decrease in frequency at *Tyro3^−/−^* synapses (Figure 2H). There was no loss in the induction of hippocampal long-term potentiation (LTP) in *Tyro3^−/−^* versus WT synapses (Figure 2I).

We also monitored the amplitude and frequency of both EPSCs and mEPSCs in the inter-connected synaptic networks that develop between mouse hippocampal neurons in culture (see Methods). We measured a substantial drop in the average EPSC amplitude in *Tyro3^−/−^* hippocampal cultures relative to WT cultures (Figure 2J), which was larger than the sEPSC amplitude drop seen in CA3 to CA1 synapses in hippocampal slices (Figure 2G). There was no difference between the average amplitude of mEPSCs in *Tyro3^−/−^* versus WT hippocampal cultures (Figure 2K). In marked contrast, dramatic reductions in the frequency of both EPSCs (Figure 2L) and mEPSCs (Figure 2M) were measured in cultures of neurons prepared from *Tyro3^−/−^* mutants compared to WT. Taken together, all of the above measurements indicate that *Tyro3^−/−^* glutamatergic synapses are physiologically impaired relative to their wild-type counterparts, and fire far less frequently.

### Immature ultrastructure of *Tyro3*^−/−^ synapses

**Figure 3:**
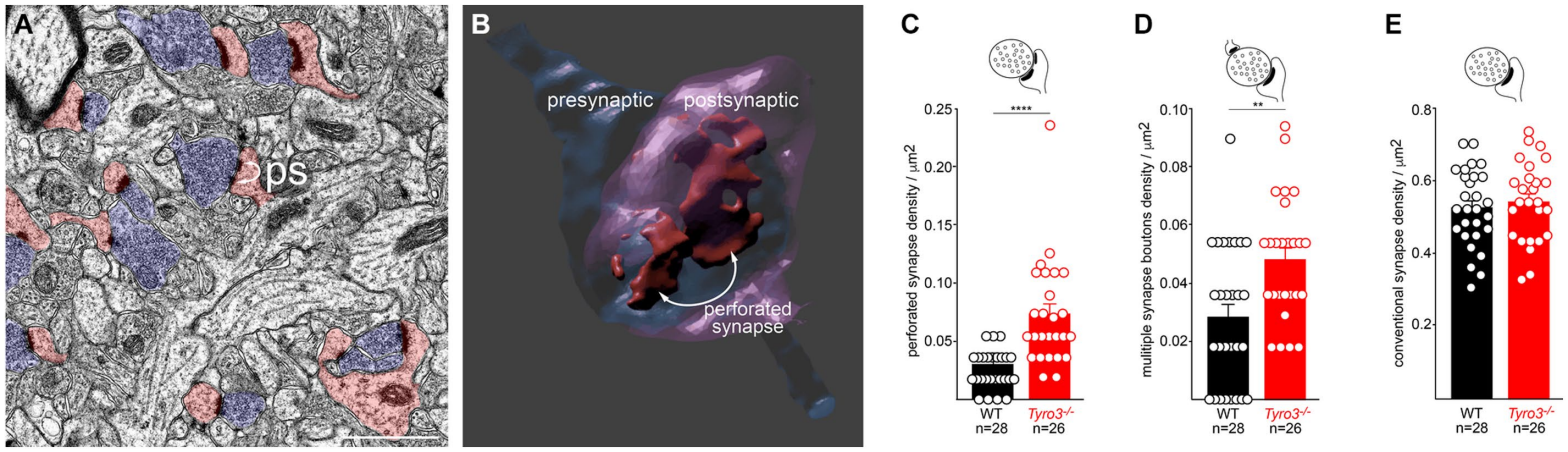
Immature ultrastructure of *Tyro3^−/−^* synapses. **(A)** Representative negatively stained transmission electron microscopic image of the stratum radiatum from the *Tyro3^−/−^* hippocampus at P60. Synaptic vesicle-filled pre-synaptic axonal boutons are blue. Dendritic spines with post-synaptic densities are pink. Some of these display a non-contiguous post-synaptic density, or ‘perforated synapse’ (ps; curved arrows). **(B)** 3D image of a perforated synapse reconstructed from serial EM sections, showing a discontinuous post-synaptic density. **(C–E)**, Quantification of the incidence of perforated synapses **(C)**, multiple synapse boutons (**D**, MSB), and conventional synapses **(E)** in WT versus *Tyro3^−/−^* stratum radiatum, from P60 EM images as in **(A)**. The total number of fields quantified (n) from 3 separate mice of each genotype (6 sections per mouse) is indicated: for WT, 28 quantifications were performed from 18 sections, and for *Tyro3^−/−^*, 26 quantifications were performed from 18 sections. *p*-values: ** <0.01, **** <0.0001, Mann–Whitney test. Scale bar: 1 μm.

Given these results, we used transmission electron microscopy (EM) to examine the ultrastructure of synapses in the stratum radiatum (boxed in Figure 1D), the synaptic region in which our hippocampal slice recordings were performed, in both adult WT and *Tyro3^−/−^* mice. We analyzed multiple slices from three mice of each genotype. In EM images of osmium tetroxide-stained ultrathin sections, synapses are readily identified by the apposition of synaptic-vesicle-filled pre-synaptic axonal varicosities (blue boutons in Figure 3A) to post-synaptic dendritic spines (pink dendritic structures in Figure 3A; Harris and Weinberg, 2012). We quantified the presence of perforated synapses (ps in Figure 3A), which are discontinuous distributions of electron-dense post-synaptic densities, since these structures have been found to be more prevalent in developing, remodeling, and immature synaptic connections (Luscher et al., 2000; Connor et al., 2006; Nicholson et al., 2006; Nicholson and Geinisman, 2009). Three-dimensional reconstructions of serial sections (example in Figure 3B) revealed that perforated synapses typically displayed complex electron-dense post-synaptic densities that were split into multiple distinct domains. We found that the incidence of these perforated synapses was 2.7-fold greater in the stratum radiatum of *Tyro3^−/−^* as compared to WT mice (Figure 3C). Multiple synapse boutons (msb), endings that make synaptic contacts with more than one spine, have similarly been found to be more prevalent in immature synapses. Correspondingly, we quantified 1.8-fold higher levels of multiple synapse boutons in the *Tyro3^−/−^* as compared to the WT hippocampus (Figure 3D). Importantly, the density of morphologically conventional synapses – measured at ~0.52/μm^2^ - was unchanged between these two genotypes (Figure 3E). Note that even in the *Tyro3^−/−^* hippocampus, these conventional synapses outnumber perforated synapses by approximately 10 to 1. Although their cytology is apparently normal in the EM, the physiological data of Figure 2 indicate that these *Tyro3^−/−^* ‘conventional’ synapses fire less frequently than their WT counterparts.

### Regulation of AMPA receptor membrane insertion by Tyro3

A key event in the maturation of glutamatergic synapses in the hippocampus and cortex is the translocation of the AMPA glutamate receptor (AMPAR) subunit GluA2 from intracellular vesicular compartments to the surface plasma membrane at the synaptic cleft (Huganir and Nicoll, 2013; Hanley, 2014). This translocation leads to a switch in the preponderance of glutamate receptor subtypes present at excitatory synapses - from N-methyl-D-Aspartate (NMDA)-type receptors and non-GluA2-containing AMPARs, both of which form calcium-permeable channels - to NMDA-type receptors and GluA2-containing receptors, the latter forming calcium-impermeable channels (Kumar et al., 2002; Brill and Huguenard, 2008; Huganir and Nicoll, 2013). This switch in turn drives synapse maturation by inhibiting calcium-dependent plasticity pathways (Jia et al., 1996; Kumar et al., 2002; Brill and Huguenard, 2008; Traynelis et al., 2010; Henley and Wilkinson, 2016). Putative loss-of-function variants in the human GluA2 (*GRIA2*) gene have been associated with several neurodevelopmental disorders (Salpietro et al., 2019).

**Figure 4:**
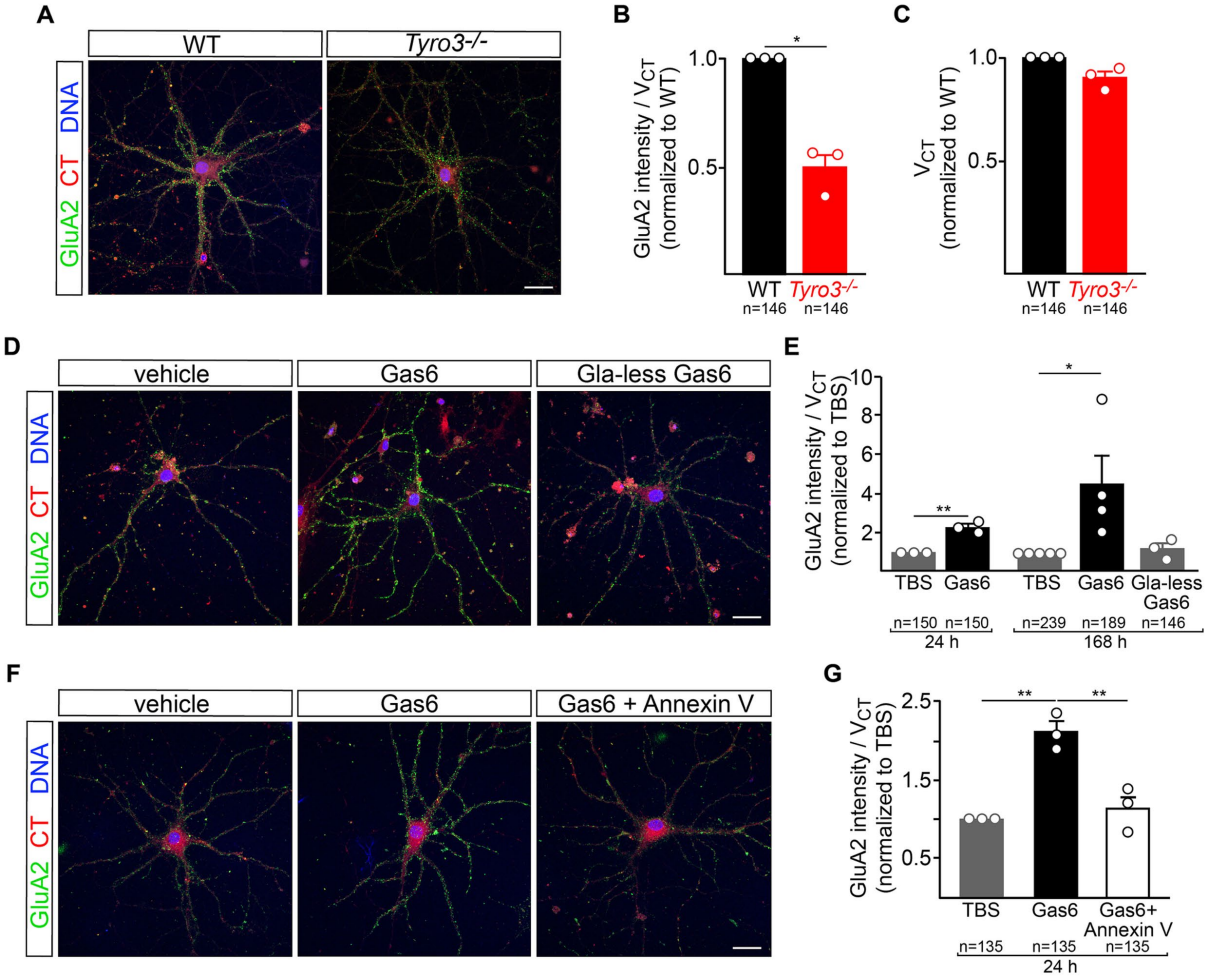
Tyro3 activation drives membrane insertion of GluA2. **(A)** Representative images of GluA2 surface expression, detected by 30 min incubation with a GluA2 antibody (Blanco-Suarez et al., 2018), in cortical neurons prepared from WT versus *Tyro3^−/−^* mice, at 18DIV. **(B)** Imaris quantification of GluA2 surface expression per cell volume (V_CT_, quantified by CellTracker Red staining), normalized to WT, in WT versus *Tyro3^−/−^* neurons. In this panel, and in panels **(C,E,G)**, *n* is the total of number of neurons analyzed from 3 to 5 separate culturing experiments (of the indicated genotypes) for each of the various experiments, with the number of culturing experiments indicated by the number of plotted data points. The value of these plotted points is the average of all cells analyzed in the same culturing experiment. **(C)** Imaris quantification of cell volume (V_CT_) normalized to WT in WT versus *Tyro3^−/−^* neurons. **(D)** Representative images of GluA2 surface expression in cortical neurons at 18DIV treated for 7 days with vehicle control (TBS, left), 20 nM recombinant Gas6 (middle), or 20 nM recombinant Gla-less Gas6, which binds but does not activate Tyro3 (right). **(E)** Imaris quantification of GluA2 surface expression per cell volume (V_CT_) in neurons incubated with the indicated reagents for the indicated times, normalized to TBS. **(F)** Representative images of GluA2 surface expression in cortical neurons treated for 1 day with vehicle control (TBS, left), 20 nM recombinant mouse Gas6 (middle), or 20 nM recombinant mouse Gas6 plus 100 nM Annexin V (right). **(G)** Imaris quantification of GluA2 surface expression per cell volume (V_CT_) in neurons incubated with the indicated reagents for 24 h, normalized to TBS. *p*-values: * <0.05, ** <0.01. Scale bars: 20 μm.

We monitored the appearance of GluA2 on the surface of live cultured mouse cortical neurons with a GluA2 antibody (Blanco-Suarez et al., 2018) added to the culture medium, and quantified surface GluA2 fluorescence signals with Imaris software, as described previously (see Methods). We carried out our analyses with neuronal cultures at 18–20 DIV, when all components of the TAM signaling pathway are present (Supplementary Figures S1A–D). We first compared surface GluA2 expression in WT versus *Tyro3^−/−^* cortical neurons. We quantified a 50% reduction in GluA2 expression, normalized to cell volume (quantified with CellTracker Red), in mutant versus WT cells (Figures 4A,B). It is important to note that mutation of *Tyro3* did not result in any change in cortical neuron volume (Figure 4C).

We then examined the ability of recombinant mouse Gas6, which acts as a potent Tyro3 ligand, to drive surface plasma membrane insertion of GluA2 in WT cortical neurons. We found that 7 days treatment with 20 nM Gas6 led to a 4-fold increase in surface GluA2, while a 24 h incubation resulted in a twofold increase (Figures 4D,E). The TAM RTKs are the only receptor system through which Gas6 is known to signal (Lew et al., 2014), and cortical neurons do not express Axl or Mer. As noted above, TAM signaling is dependent on the binding of the amino-terminal Gla domain of Gas6 (and Pros1) to the phospholipid PtdSer, which is externalized on the plasma membrane surface of cultured neurons (Figure 1G). When we treated cultured cortical neurons with recombinant Gla-less Gas6, an amino-terminally truncated protein that binds to TAM receptors with normal affinity but that cannot bind PtdSer and cannot normally activate the kinase activity of Tyro3 (Lew et al., 2014), we found that it was incapable of driving GluA2 insertion into the neuronal plasma membrane (Figures 4D,E). Consistent with all of these effects, we found that 24 h pre-treatment with 100 nM recombinant Annexin V, a high-affinity PtdSer binding protein (Tait et al., 1989; Fadok et al., 1992), completely blocked the ability of full-length Gas6 to stimulate the cell surface expression of GluA2 (Figures 4F,G). These results are consistent with the display of PtdSer on a pre-synaptic neuronal plasma membrane that is facing a Tyro3-containing post-synaptic membrane, with Gas6 interposed as a bridge between these two membranes.

### Behavioral consequences of *Tyro3* mutation

The above results demonstrate that Tyro3 activation promotes the insertion of GluA2 AMPAR subunits into the neuronal plasma membrane, that *Tyro3^−/−^* synapses *in vivo* are GluA2-deficient, and that these synapses are physiologically and ultrastructurally immature. These observations are consistent with the finding that *Tyro3^−/−^* synapses in the hippocampus showed no loss of plasticity relative to WT synapses upon induction of LTP (Figure 2I), and with previous work that has demonstrated that hippocampal LTP is dramatically enhanced at synapses that lack all GluA2 (Jia et al., 1996; Mainen et al., 1998). Given these findings, we assessed the extent to which *Tyro3* gene deletion effected adult (P84–98) mouse behavior in two different assays. Prior to carrying out these assays, we first used continuous video monitoring in open fields (Seibenhener and Wooten, 2015) (see Methods) to ascertain that both overall mouse locomotion and anxiety behavior were unaffected by the mutation of *Tyro3* (Supplementary Figures S2A,B). One physiological feature that was increasingly affected with increasing age was body weight, as WT mice were 10% heavier than *Tyro3^−/−^* mice by 7 months of age (Supplementary Figure S2C). This small effect did not contribute to the behavioral differences described below, since spatial memory and fear conditioning tests were performed at 3 months, when the weight of WT and *Tyro3^−/−^* mice was not statistically different (Supplementary Figure S2C). As discussed below, however, it may be relevant to potential Tyro3 regulation of feeding behavior.

**Figure 5:**
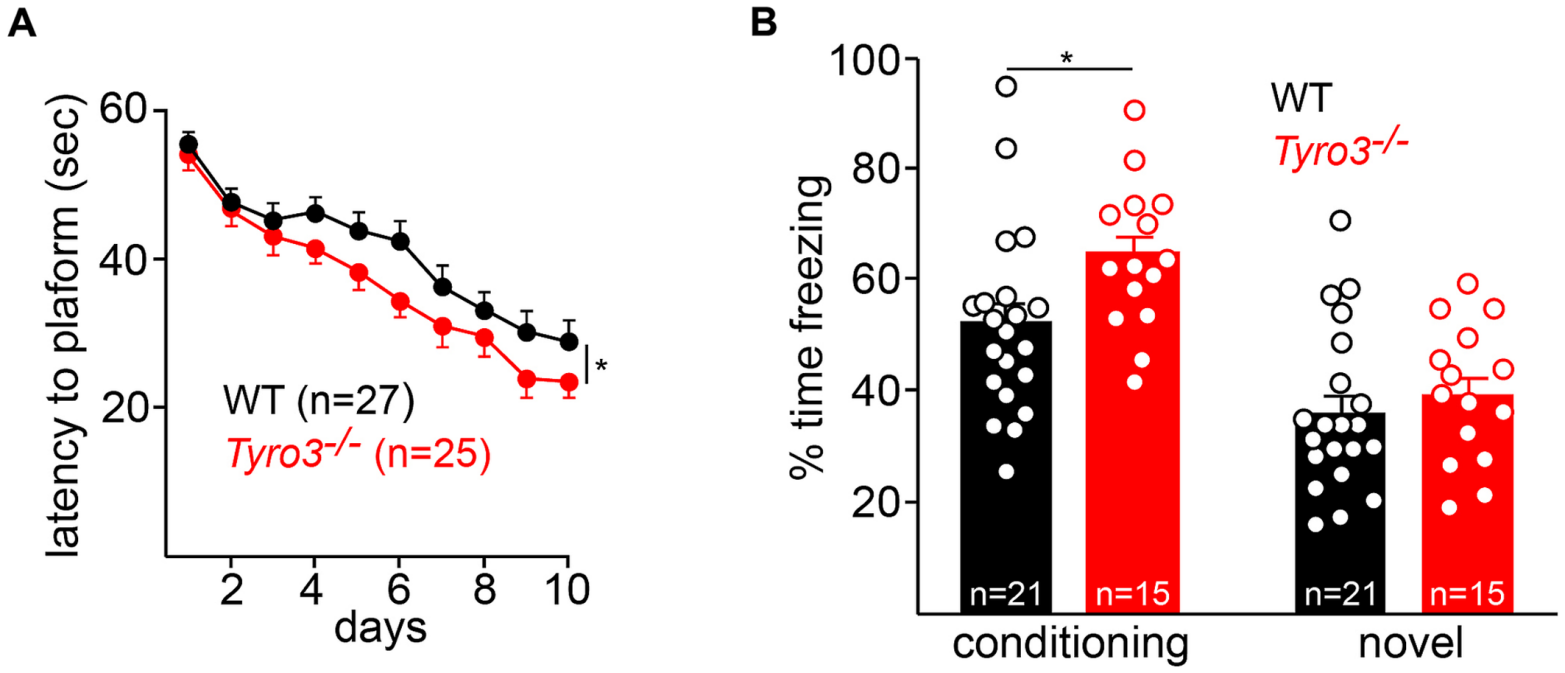
Behavioral improvements in *Tyro3^−/−^* mice. **(A)** Adult *Tyro3^−/−^* mice take less time to detect the submerged platform in a Morris water maze test (see Methods) than their WT counterparts beginning at 3 days of testing. **(B)** When returned to the training (‘conditioning’) context, *Tyro3^−/−^* mice exhibit enhanced memory recall, as monitored by elevated freezing behavior (left). No difference in freezing is detected when mice are placed in a novel context (right). *n* is total number of mice tested per genotype. *P*-values: * <0.05.

We assessed spatial memory acquisition using a standard Morris water maze test (Morris, 1984; Vorhees and Williams, 2006) (see Methods). In the spatial acquisition phase of this assay, mice are trained over the course of 10 days to locate and remember the position of a platform submerged just below the surface of an opaque pool of water (see Methods). We found that *Tyro3^−/−^* mice performed better than their WT counterparts at this task. After 3 days of initial training, the mutant mice consistently – across days 4 through 10 of assay – took significantly less time to locate the submerged platform than WT mice (Figure 5A).

This effect, while statistically significant, was nonetheless small. We additionally performed a contextual fear conditioning test in which we quantified the ability of mice to remember the association between a context and an aversive mild electrical stimulus (Kitamura et al., 2014) (see Methods). After a 10-min period of free exploration on the first day, mice were returned to the same context on the second day, where they were allowed 42 s to establish an association between electric shocks (2 s, 0.5 mA) and the environment. On the third day, freezing behaviors were monitored in both the conditioning context and a novel context. We found that *Tyro3^−/−^* mice displayed a significantly enhanced memory recall in the conditioning context, as quantified by freezing time, relative to WT (Figure 5B). No difference in freezing time was observed between genotypes in a novel context. Thus, in both of these behavioral assays, the *Tyro3^−/−^* mutants exhibited improved performance consistent with increased synaptic plasticity.

### A model for Tyro3 activity at glutamatergic synapses

**Figure 6:**
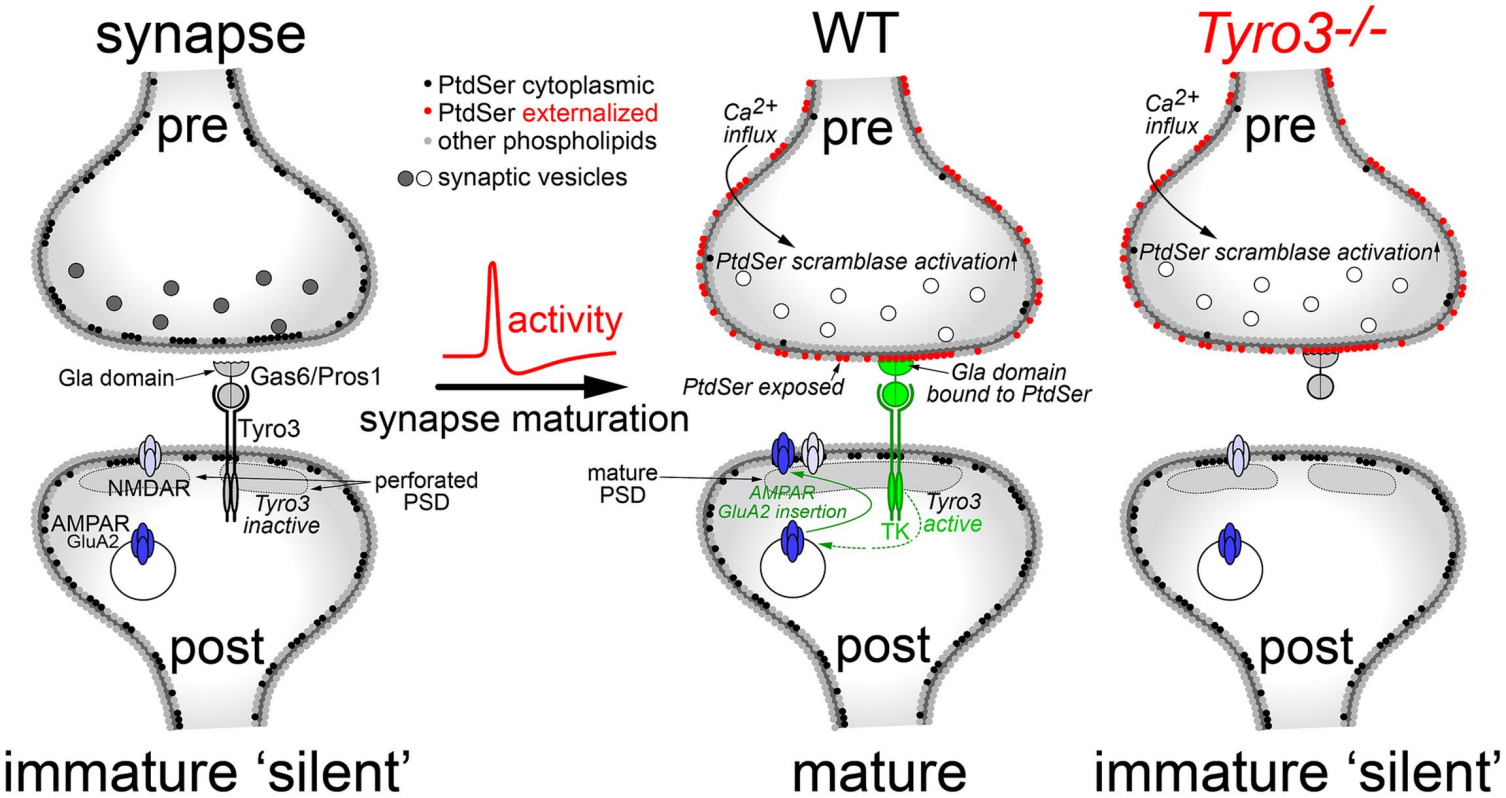
A model for Tyro3-driven synaptic maturation. In a developing, electrically silent synapse (left), PtdSer is confined to the inner leaflet of the pre-synaptic plasma membrane (black circles) by the action of flippases, which operate in all cells. The lack of PtdSer externalization means that the Gla domains of the extracellular Tyro3 ligands Gas6 and Pros1 cannot bind this phospholipid, which is required for Tyro3 activation. In the absence of Tyro3 activation, many of the GluA2 AMPA receptor subunits required for mature synapses (AMPAR, dark blue) remain in internal endosomes. These are not transported to the plasma membrane surface, which largely contains NMDA-activated glutamate receptors (NMDAR, light blue) and non-GluA2-containing AMPA receptors, both of which are calcium permeable. As a group, these immature synapses also display an elevated incidence of cytologically disorganized and non-contiguous, or perforated, post-synaptic densities (PSDs). Upon the arrival of an action potential at the pre-synaptic terminal, Ca^2+^ flows into the terminal resulting in the activation of Ca^2+^-dependent scramblases (middle). This scramblase activation leads to PtdSer externalization on the pre-synaptic plasma membrane surface (red circles), where it can bind to the Gas6/Pros1 Gla domains. Coincident Gas6/Pros1 binding to PtdSer and Tyro3 strongly activates the Tyro3 tyrosine kinase (TK), which promotes both the transport of GluA2 subunits to the post-synaptic plasma membrane surface and the phosphorylation of cytoskeletal proteins (Happonen et al., 2023) that results in the consolidation of the PSD. In the absence of Tyro3 (right), PtdSer is still externalized, but post-synaptic Tyro3 signaling cannot occur. Gray and white synaptic vesicles represent transmitter-filled and transmitter-released vesicles, respectively.

All of the above results are consistent with a model in which post-synaptic activation of the catalytic activity of the Tyro3 tyrosine kinase drives the maturation of glutamatergic synapses by promoting the plasma membrane insertion of GluA2-containing AMPA receptors and the consolidation of the post-synaptic density (Figure 6). As noted above, GluA2 insertion is required for synaptic stabilization and maturation because it renders heteromultimeric AMPARs Ca^2+^-impermeable. Our model requires pre-synaptic externalization and plasma membrane display of the phospholipid PtdSer, which is activated by depolarization of pre-synaptic terminals (Figure 6, center). As discussed below, PtdSer externalization is carried out by two large families of scramblase enzymes, one of which is controlled (activated) by Ca^2+^ (Suzuki et al., 2010; Whitlock and Hartzell, 2017; Whitlock, 2021). Since calcium enters pre-synaptic terminals through voltage-activated calcium channels upon the arrival of an action potential (Bean, 2007), and since multiple Ca^2+^-dependent scramblases are widely expressed in the brain (Suzuki et al., 2013b), Ca^2+^ entry would activate these scramblases. The resulting surface expression of PtdSer would provide binding sites for the Gla domains of the TAM ligands Gas6 and Pros1, without which effective Tyro3 activation cannot occur. Post-synaptic Tyro3 engagement is in turn known to activate a series of downstream kinases that have been found to be required for GluA2 translocation from intracellular stores to the post-synaptic plasma membrane surface (Chan et al., 2011; Anggono et al., 2013). At a *Tyro3^−/−^* synapse, pre-synaptic PtdSer externalization still occurs upon depolarization (Figure 6, right), but post-synaptic insertion of GluA2 cannot occur. This model provides a fundamentally new mechanism whereby coupled pre- and post-synaptic activity drives the maturation of synapses (Figure 6).

## Discussion

In Hebbian learning, neurons that fire together, wire together (Hebb, 1949; Morris, 1999). Our results suggest that post-synaptic Tyro3 signaling provides one molecular mechanism through which coordinated electrical activity drives Hebbian synaptic consolidation. Our model for Tyro3 action in the stabilization and maturation of post-synaptic dendritic spines requires the externalization of the phospholipid PtdSer on pre-synaptic axonal boutons (Figure 6). As noted above, this externalization can only be achieved through the action of large 10-transmembrane-domain enzymes known as scramblases, since the inner-to-outer leaflet exchange of any phospholipid has a high energy barrier (15–50 kcal/mol) and cannot occur spontaneously (Kornberg and McConnell, 1971; Whitlock and Hartzell, 2017). These scramblases fall into two distinct families – the TMEM16 family, also referred to as Anoctamins, which are encoded by 10 human genes (Suzuki et al., 2010, 2013b; Whitlock and Hartzell, 2017; Watanabe et al., 2018); and the XKR family, which encompasses 9 human genes (Nagata, 2018; Lemke, 2019). We favor the possibility that TMEM16/Anoctamin proteins (gene names *Ano1-10*) may operate in our model at pre-synaptic terminals, since these enzymes are activated by the intracellular Ca^2+^ that rises dramatically with the arrival of an action potential, and since *Ano8*, *Ano10*, and especially *Ano6* (*TMEM16F*) mRNAs are highly and widely expressed by CNS neurons (Suzuki et al., 2013b; Zhang et al., 2014). Our results indicate that electrically-active cortical neurons in culture display externalized PtdSer, and that calcium entry into these cells triggered by brief exposure to a calcium ionophore results in exuberant PtdSer expression on the membrane surface (Figure 1G). The TMEM16F enzyme expressed by CNS neurons is the most widely studied of the Ca^2+^-regulated scramblases (Soulard et al., 2020). However, we do not exclude a role for XKR family scramblases, since elevated levels of activated (cleaved) caspases, notably cleaved caspase 3, have been detected in proteomic analyses of murine synaptosomes (Gyorffy et al., 2018), and caspase cleavage is required for the activation of the XKR scramblases XKR4, 8, and 9 (Nagata and Segawa, 2021).

The proteins that bridge PtdSer on the pre-synaptic membrane to Tyro3 on the post-synaptic membrane are the TAM ligands Gas6 and Pros1. The *Gas6* gene is very widely and constitutively expressed in neurons across many areas of the postnatal and adult CNS, including neurons of the neocortex, and in pyramidal neurons across all fields of the hippocampus, with high expression in CA3 (Prieto et al., 1999). All CNS microglia also express abundant *Gas6* mRNA (Imm Gen, 2016). Neuronal expression of the *Pros1* gene is much more restricted, with *Pros1* mRNA and protein detected in neural stem cells, but prominent CNS expression of *Pros1* mRNA is detected in endothelial cells, microglia, and astrocytes (Imm Gen, 2016; Zelentsova et al., 2017; Zelentsova-Levytskyi et al., 2017; Happonen et al., 2023) (see footnote 2). Pros1 is also present at ~300 nM in the CNS circulation (Burstyn-Cohen et al., 2009). Together, these observations indicate that the ligands required to activate Tyro3 are very widely expressed in the brain, and can in principle be delivered to neurons from multiple sources.

In general, the electrophysiological and ultrastructural properties of glutamatergic synapses in the *Tyro3^−/−^* hippocampus and cortex are characteristic of synaptic pairings that have stalled at a late stage of differentiation. These synapses are immature by multiple measures and display modification plasticities that are consistent with diminished synaptic membrane expression of the GluA2 subunit of ionotropic glutamate receptors. For example, the observation that CA3-to-CA1 LTP is not lost in *Tyro3^−/−^* hippocampal slices is consistent with the deficit in surface GluA2 expression that we detected in *Tyro3^−/−^* neurons (Figures 4A,B), since hippocampal slices prepared from *GluA2^−/−^* mice, which lack all GluA2, display enhanced LTP (Jia et al., 1996; Gerlai et al., 1998). The modest behavioral improvements in spatial memory and fear conditioning that we detected in the *Tyro3^−/−^* mice are also consistent with synaptic connections that are plastic. Similar behavioral improvements have been documented upon loss-of-function mutation of many ‘memory suppressor genes’ (Abel et al., 1998; Lee and Silva, 2009; Noyes et al., 2021). Again, we posit that an important component of *Tyro3^−/−^* plasticity is the reduced expression of GluA2-containing glutamate receptors at the post-synaptic membrane surface. Although *Tyro3^−/−^* synapses are deficient in these receptors, they presumably have a normal complement of the NMDA receptors that populate immature synapses (Ramoa and McCormick, 1994; Kim et al., 1995; Lopez de Armentia and Sah, 2003) and normal glutamate release, although we did not test either of these points in this work. Our electrophysiological results clearly indicate that the *Tyro3^−/−^* stratum radiatum has fewer active synapses, yet the number of synapses we measured by EM was either the same (for conventional synapses) or increased (for perforated synapses). We do not currently have an explanation for this difference.

An additional feature of the *Tyro3^−/−^* mice that may be relevant to an influence of this RTK on later behavior (after the time period of our assays) is the modest but clear weight loss that we observed as the knockouts aged (Supplementary Figure S2C). A mutation in the signal sequence of the mouse *Tyro3* gene (R7W), which resides within the critical interval that defines the *anorexia* (*anx*) mutation in *anx*/*anx* mice (Maltais et al., 1984), but is not the *anx* mutation itself, has been shown to be an important genetic modifier of the phenotype exhibited by these anorexic mice (Kim et al., 2017). Although they are able to eat and have full access to milk, *anx/anx* mice die at 3–5 weeks after birth due to starvation and emaciation (Nilsson, 2019). Tyro3 is expressed in regions of the hypothalamus (e.g., the arcuate nucleus) that control feeding behavior; and transgenic CNS expression of a WT *Tyro3* transgene, but not an *R7W Tyro3* transgene, has been found to double the weight and lifespans of *anx/anx* mice (Kim et al., 2017).

## Data availability statement

The original contributions presented in the study are included in the article/Supplementary material, further inquiries can be directed to the corresponding author.

## Ethics statement

The animal study was approved by Salk Institute Animal Care and Use Committee. The study was conducted in accordance with the local legislation and institutional requirements.

## Author contributions

SM: Conceptualization, Data curation, Formal analysis, Investigation, Methodology, Supervision, Writing – review & editing. LF: Conceptualization, Data curation, Writing – review & editing, Investigation, Methodology. PB: Formal analysis, Investigation, Methodology, Writing – review & editing. SS: Data curation, Investigation, Methodology, Writing – review & editing. YZ: Data curation, Investigation, Writing – review & editing. KH: Data curation, Writing – review & editing. SN: Methodology, Writing – review & editing, Data curation. FG: Methodology, Writing – review & editing. GL: Conceptualization, Data curation, Formal analysis, Funding acquisition, Project administration, Resources, Supervision, Writing – original draft, Writing – review & editing.

## Footnotes

^1^blender.org

^2^https://endotheliomics.shinyapps.io/ec_atlas/
