# Supplementary material for: Tyro3 promotes the maturation of glutamatergic synapses
Source: Front Neurosci. 2024 Feb 12;18:1327423. doi: 10.3389/fnins.2024.1327423 (PMC10894971; doi:10.3389/fnins.2024.1327423)
Supplement: Supplementary file 1 [file Table_1.docx]

**Supplementary Material for**

**Tyro3 promotes the maturation of glutamatergic synapses**

**Sheng Miao, Lawrence Fourgeaud, Patrick G. Burrola, Shani Stern, Yuhan Zhang, Kaisa E. Happonen, Sammy Weiser Novak, Fred H. Gage, and Greg Lemke**

**Figure Legends**

**Supplemental Figure S1. PtdSer expression on the membrane surface of maturing cortical neurons *in vitro*. A**, Representative western blot analyses illustrating maturation of cortical neurons prepared from E18 mouse embryos and cultured for the indicated days *in vitro* (DIV), as monitored by the expression of the indicated proteins. Gapdh is a loading control. **B**, Representative examples of PtdSer externalization on cortical neurons over time, visualized with pSIVA binding (green) to neurons cultured for the indicated DIV. Three example fields are shown for each time point. CT, CellTracker dye (red); DNA, DAPI (blue). **C**, PtdSer on the surface of two cortical neurons at 20 DIV. Left, pSIVA. Middle, pSIVA with CT and DAPI. Note healthy (non-apoptotic) nucleus (inset). Right, boxed area in middle panel is enlarged. **D**, PtdSer (pSIVA, green) on the surface of cortical neurons at 20 DIV (Left), stained post-pSIVA with an antibody to synaptophysin (synapto, red). Nuclei visualized with DAPI (blue) and cell volumes with CellTracker (CT, gray). Boxed area is enlarged in the individual channels (pSIVA, synapto, merged pSIVA/synapto, and CT) at right. Circled areas highlight areas of co-localization between pSIVA labeling of PtdSer and staining for synaptophysin. Scale bars (**B** - **D**): 20μm.

**Supplemental Figure S2. *Tyro3^-/-^* mice display normal locomotion and anxiety behavior. A**, Continuous video monitoring indicates that distance traveled within open field chambers over 20 min is not different between WT and *Tyro3^-/-^* mice. **B**, Anxiety in laboratory mice is marked by preferential occupancy near the walls of open field chambers with limited exploration of the center. Continuous video monitoring indicates that WT and *Tyro3^-/-^* mice spend an equivalent fraction of time in chamber centers. **C**, Body weight of WT versus *Tyro3^-/-^* mice at the indicated ages. P value: **** <0.0001. For all panels, n is the number of mice analyzed.
