# Supplementary figures and images for: Tyro3 promotes the maturation of glutamatergic synapses
Source: Front Neurosci. 2024 Feb 12;18:1327423. doi: 10.3389/fnins.2024.1327423 (PMC10894971; doi:10.3389/fnins.2024.1327423)

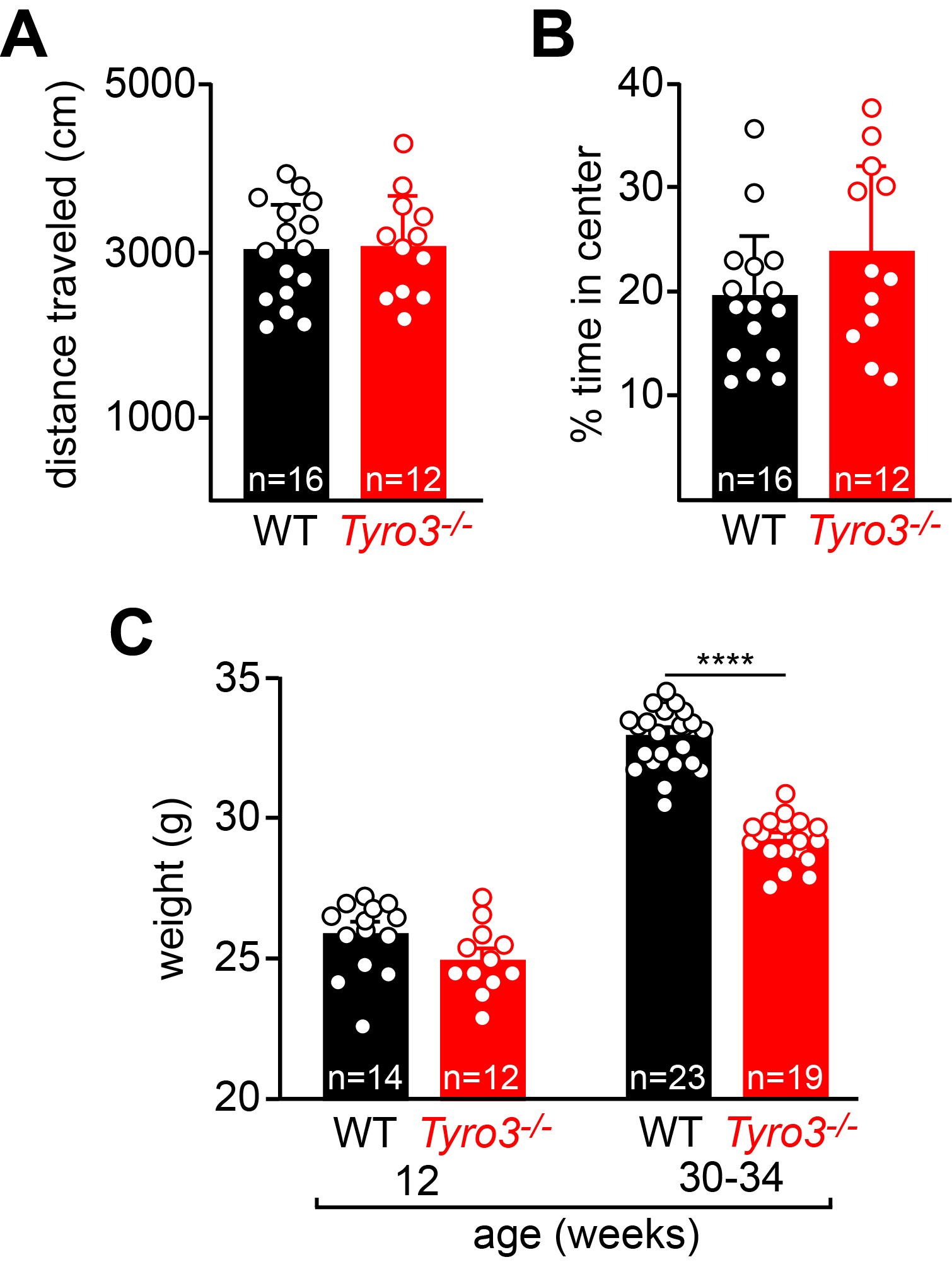

Supplement: Supplementary file 2 [file Figure_1.JPEG]

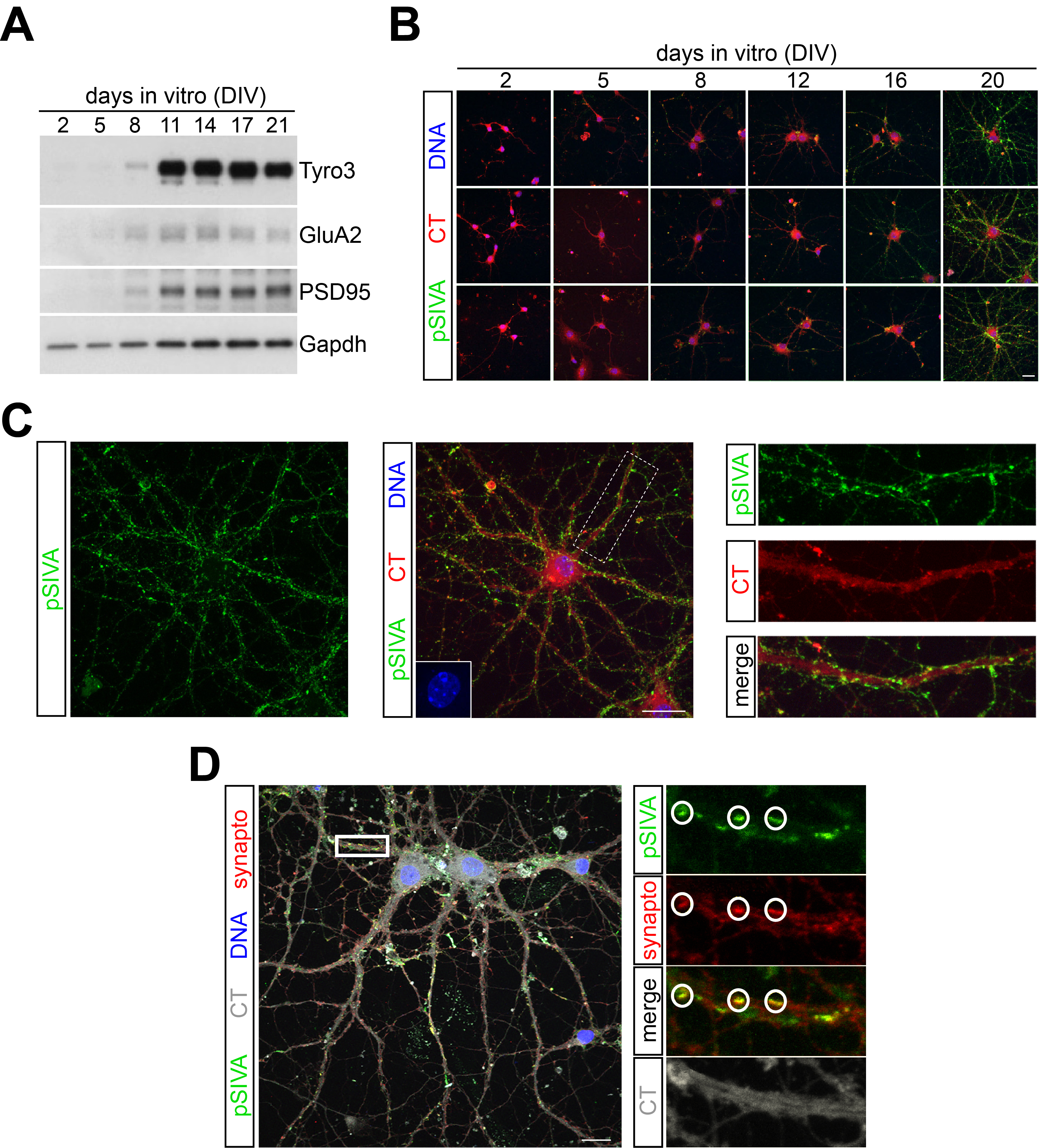

Supplement: Supplementary file 3 [file Figure_2.JPEG]
